# Not Only Top-Down: The Dual-Processing of Gender-Emotion Stereotypes

**DOI:** 10.3389/fpsyg.2020.01042

**Published:** 2020-05-26

**Authors:** Wen-long Zhu, Ping Fang, Hui-lin Xing, Yan Ma, Mei-lin Yao

**Affiliations:** ^1^Beijing Key Laboratory of Applied Experimental Psychology, Faculty of Psychology, Beijing Normal University, Beijing, China; ^2^Beijing Key Laboratory of Learning and Cognition, Department of Psychology, Capital Normal University, Beijing, China

**Keywords:** gender-emotion stereotypes, face perception, emotional expressions, dual-processing, process-dissociation procedure, affordance management

## Abstract

Is gender-emotion stereotype a “one-hundred percent” top-down processing phenomenon, or are there additional contributions to cognitive processing from background clues when they are related to stereotypes? In the present study, we measured the gender-emotion stereotypes of 57 undergraduates with a face recall task and found that, regardless of whether the emotional expressions of distractors were congruent or incongruent with targets, people tended to misperceive the fearful faces of men as angry and the angry faces of women as fearful. In particular, there was a significantly larger effect in the distractor-incongruent condition. The revised process-dissociation procedure analysis confirmed that both automatic and controlled processing have their own independent effects on gender-emotion stereotypes. This finding supports a dual-processing perspective on stereotypes and contributes to future research in both theory and methodology.

## Introduction

Stereotype is one of the core issues in social and psychological fields because of its significant impact on information processing in social cognition. During the past several decades, researchers have predominantly examined stereotype from a top-down processing perspective, in which the stereotypical response tendency is commonly considered to be an automatic consequence of cognitive processing (McCrea et al., 2012). That is, whenever we encounter or merely think of the members of a specific social category, such as race, gender or age, stereotypes are activated automatically and then reflected imperceptibly in our thoughts and behaviors (Kornadt, 2016; Cooley et al., 2018). But, as demonstrated by previous studies, a controlled processing component has been found in some stereotype-related thought, in addition to the well-known automatic processing one (Devine, 1989; Johnston and Coolen, 1995; Blair and Banaji, 1996), and this dual-processing mechanism may also exist in gender-emotion stereotype (Neel et al., 2012). In this study, we address the above issue using a specific analytic procedure to examine the dual-processing mechanism of gender-emotion stereotype.

### Gender-Emotion Stereotype

There are many phenomena tied with gender-related stereotypes in daily life, such as gender stereotypes reflected in the aspects of academic setting (Law, 2018; Muntoni and Retelsdorf, 2018) or career development (Heilman, 2012; Cadaret et al., 2017). For example, boys are believed to be outstanding in mathematics but weak in language learning, whereas girls are believed to be exactly the opposite and are consequently constrained in the choice and progress of math-related work. In particular, gender-emotion stereotype is regarded as one of the most common but complicated stereotypes (Shields, 2013; Brody et al., 2016; Harris et al., 2016).

Numerous studies have found that people tend to perceive women as more “emotional” than men, regardless of the intensity of the experience (Boucher et al., 2015) or the frequency and skill of the expression (Adams et al., 2015). It has also been found that the effect size of the belief about gender differences in emotional expression is two to four times larger than other effect sizes concerning personality traits and cognitive abilities (Brescoll, 2016). Nevertheless, more subtle connections still exist between gender and emotion with regard to specific emotions. It is generally believed that sadness, happiness, fear, jealousy, surprise, embarrassment, shame, and guilt occur more frequently in women, whereas other emotions, such as anger, contempt, disgust, and pride, are viewed as typically “masculine emotions” (e.g., Hess et al., 2000; Plant et al., 2000; Safdar et al., 2009). For example, Plant and her colleagues manipulated the gender characteristics of faces to present the same face as either male or female and then asked participants to rate the expressed emotions (Plant et al., 2004). They found that feminine faces were rated as significantly sadder than masculine faces, reflecting the stereotypical correspondence between gender and emotion. Adams et al. (2012) found that neutral female faces were rated as more fearful and happier but less angry than neutral male faces.

However, the direction of bias does not always match gender-emotion stereotypes in a straightforward way. Hess et al. (2004) have also found that, when the same face appeared as either male or female, angry female faces were rated as angrier than angry male faces, whereas happy male faces were rated as happier than happy female faces. As for the contradictory findings, Hess et al. (2004) ascribed the above results to their having controlled for facial appearance (i.e., dominance vs. affiliation), which acted as a mediator. Another explanation is that the opposite effect was due to the conflict between the displayed stimuli and the expectations for expressions of different genders. Specifically, stereotypical expectations might amplify the interpretation of expressive cues, such as “women don’t usually show anger, so that angry-looking woman must be really angry” (Brody et al., 2016). As a social cognition generated from each particular cultural norm, gender-emotion stereotype and its form and intensity may also show a certain cultural specificity. For example, previous studies have found that sadness is more appropriate for women to express in Canada, the United States, and Japan (Safdar et al., 2009), whereas it is more appropriate for men to express in Singapore (Moran et al., 2013). Thus, it is necessary to examine the specific influence of gender-emotion stereotypes on individual’s perception and judgment in particular culture.

### Dual-Processing of Stereotypes

Since stereotype-related thought mainly involves the extraction and externalization of prior schemas and scripts, it has usually been considered to be an automatic processing phenomenon (e.g., Banaji and Hardin, 1996). McCrea et al. (2012) found that individuals with a higher construal level (i.e., a top-down, global, abstract processing) were more prone than individuals with a lower construal level (i.e., a bottom-up, local, concrete processing) to evaluate themselves and others in correspondence with stereotypes. There is also evidence from event-related potentials (ERPs) that sentences with a terminal word violating gender stereotypes elicit a greater anterior N400 response and left anterior negativity (LAN), suggesting the activation of stereotypes in an implicit task without any priming stimuli (Proverbio et al., 2017).

However, prior studies have also indicated that stereotypes may have both automatic and control processing components. Devine (1989) and Blair and Banaji (1996) found that participants who build a counterstereotype belief would inhibit the automatically activated stereotype-related thought, suggesting both automatic and controlled processes exist in stereotype priming. Johnston and Coolen (1995) manipulated the cognitive involvement and message cues, and then measured stereotype-related thought. They found an additive effect of source credibility and message strength on stereotype in low involvement condition. Concretely, as source credibility and message strength improve, stereotypical response decrease significantly, suggesting stereotype-related information could be captured and affect subsequent processing and its results. Above results all support a dual-processing model of stereotype to a certain degree.

Payne (2001) initially introduced the process-dissociation procedure (PDP) to confirm the independent contribution of both automatic and controlled processing to race-related stereotypes in the weapon identification task (WIT). The effectiveness of PDP for evaluating dual-processing in stereotype-related tasks has been verified in subsequent studies (Huntsinger et al., 2009, 2010). The fundamental idea of the PDP analysis is that automatic and controlled processes simultaneously but independently contribute to behaviors in a given task (Jacoby, 1991), and their impact on specific trials may be either congruent or incongruent. For example, in the weapon identification task, both controlled effort and automatic activation may render stereotype-congruent trials (i.e., Black people-Weapon, White people-Tool) correct. For stereotype-incongruent trials (i.e., Black people-Tool, White people-Weapon), however, automatic and controlled processes may produce opposite results. Thus, PDP analysis can estimate exactly the contributions of both automatic and controlled processing by calculating correct and incorrect results for various trials (see the specific algorithm, Huntsinger et al., 2009, 2010). Compared with other algorithms, such as the QUAD model (Conrey et al., 2005; Wang et al., 2015), PDP analysis simply parses responses into automatic and controlled components respectively in stereotype-related tasks without any additional response bias (Huntsinger et al., 2009).

In fact, gender-emotion stereotype, which is usually represented as automatic processing, may also include a controlled component that previous studies have failed to identify. Based on the above research findings and a recent theory of dual systems for feature integration (Wolfe et al., 2011), Neel et al. (2012) found an interaction between the gender-emotion stereotype and an illusory conjunction in which the features of adjacent objects are wrongly recombined under certain conditions (Becker et al., 2010). They found that, in a face recall task, when the emotional expressions of distractors were incongruent with the target faces, male faces tended to “grab” the angry expression from a neighboring face, whereas female faces disproportionately “grabbed” happiness. Moreover, these stereotypical misperceptions of emotional expressions in the incongruent condition had greater effects than in trials with congruent emotional expressions between targets and distractors, suggesting that there may exist a controlled process of gender-emotion stereotype. Unfortunately, there have been few studies focusing on the independent effect of controlled processing on gender-emotion stereotypes (Neel et al., 2012), largely because of lacking methods for appropriate analyses. This issue could be solved by further adapting PDP analysis to distinguish controlled process of gender-emotion stereotype from automatic processing.

### The Present Study

In summary, the purpose of this study was to examine gender-emotion stereotypes under Chinese cultural background, and extends our understanding beyond the previous research by disentangling the underlying mechanisms. As typical emotions focused by previous studies on gender-related stereotypes, we choose anger and fear as contrast objects, which are similar in valence and arousal and associated stereotypically with different genders respectively. The measurement and analysis of gender-emotion stereotypes were mainly realized by the face recall task (Neel et al., 2012) and PDP analysis which has been creatively revised.

Two primary research questions guided our work. First, does gender-emotion stereotypes affect the perception of both gender and emotion? Based on prior visual processing research (Atkinson et al., 2005; Karnadewi and Lipp, 2011; Young and Bruce, 2011), which have confirmed an asymmetrical connection between gender and emotion in visual processing, we hypothesized that stereotypical response may only affect emotion perception. Concretely, when instructed to recall the emotional expressions on faces with various genders, participants would display stereotypical mistakes reflecting that anger is preferably connected to male faces and fear is preferably connected to female faces. Second, is there an independent controlled processing component in gender-emotion stereotypes? We hypothesized that the above stereotypical mistakes would both appear when distractors, which were adjacent to the targets, had congruent and incongruent expressions. In particular, the incongruent condition would have stronger effects because of the extra contribution from controlled processing which could be ultimately confirmed by PDP analysis.

## Materials and Methods

### Participants

Sixty undergraduates at Capital Normal University in Beijing, who enrolled in a psychology course participated in this experiment for partial fulfillment of the course requirement^1^. 3 of them were removed either because of high error rates in excess of chance (50%) on the face recall task or excessively low computational accuracy on the interference task (over 3 standard deviations). All of the remaining participants (36 women, 21 men) were Asians, and their average age was 20.12 years (*SD* = 1.51). Participants sex did not produce main or any interactive effects, as Neel et al. (2012) study has found.

### Procedure and Materials

Participants were run one at a time in a single computer room. After signing an informed consent agreement, participants were told that they would complete a series of computer-based tasks to test out their memory capacity, reminded to respond with correct answers as quickly as possible. We then assessed the gender-emotion stereotypes in reality using a face recall task adapted from Neel et al. (2012). At the end of the experiment, participants were thoroughly debriefed and thanked for their participation. All measurements and procedures were approved by the Institutional Review Board (IRB) of the authors’ institution.

#### Target Stimuli

**FIGURE 1:**
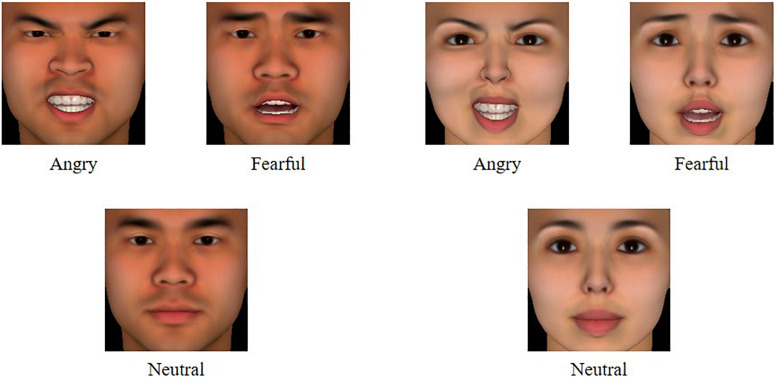
Examples of stimuli used in the critical blocks. The complete target stimuli are available from Supplementary Material.

Neel et al. (2012) findings have suggested that computer-generated faces with standardized settings are more effective for measuring stereotype than photographs of real faces, so we used stimuli created by FaceGen Modeller 3.5. We randomly generated 10 pairs of East Asian faces through the program, and each pair consisted of a male face and a female face that were equidistant from a non-sexual face on the gender dimension. We then created a neutral expression for each face and cropped the edges of them (200 × 200 pixels) to avoid the influence of hair styles on gender perception. Another 26 undergraduates were recruited to rate the degree of sexualization for each face (1 for definitely masculine, 7 for definitely feminine). According to the criteria for materials selection used by Neel et al. (2012), the faces perceived as clearly masculine should be rated less than 3, whereas the selected feminine faces should be rated more than 5^2^. Finally, 4 faces of each gender were selected for the critical trials, and another 2 faces of each gender were used in the practice materials. Angry and fearful expressions for each selected face were produced using the program’s morphing tools with the maximum of the emotion. Both were displayed open-mouthed to ensure consistent expressive intensity (see Figure 1). The neutral faces were used only in the pretest and were not displayed in the critical trials.

#### Face Recall Task

Each trial started with a fixation cross on the screen. After 150 ms, 2 faces were presented on both sides of the fixation cross, flanked by 2 single digits, which remained on the screen for 250 ms and were replaced by a post-mask. Participants were then asked to complete an interference task in which they entered the sum of the two numbers with a key press. After that, participants were given the critical task of reporting either the gender (i.e., woman or man) or the emotional expression (i.e., anger or fear) of the faces randomly presented on both sides. There were two priming conditions (i.e., distractor-congruent or distractor-incongruent) depending on the presentation mode of target stimulus. In distractor-congruent condition, the target information was congruent with the distractor (e.g., both faces are male or female when need to recall the gender) whereas in the distractor-incongruent condition the target information was incongruent with the distractor (e.g., one face is angry and the other is fearful when need to recall the emotional expression). Each trial was separated by a blank screen that lasted for 100 ms. A practice block was conducted to familiarize participants with testing interface and response mode, followed by 4 critical blocks of 32 trials each. At the end of the practice, the participants were asked whether they had already understood the experiment and whether they needed to repeat the practice. The target information (i.e., gender or emotional expression) and target positions were counterbalanced between trials, and all 16 combinations of face types (i.e., gender and emotional expression of left face × gender and emotional expression of right face, see Table 1) were equally presented.

**TABLE 1:** Mean error rates and standard deviations for all conditions.

		***M*(*SD*)**			
		**Men-Fear**	**Women-Fear**	**Men-anger**	**Women-anger**
Target with congruent distractor	Emotion	0.364 (0.186)	0.230 (0.156)	0.228 (0.144)	0.338 (0.172)
	Gender	0.250 (0.134)	0.230 (0.145)	0.241 (0.143)	0.252 (0.161)
Target with incongruent distractor	Emotion	0.478 (0.189)	0.243 (0.150)	0.268 (0.171)	0.489 (0.171)
	Gender	0.263 (0.131)	0.232 (0.146)	0.239 (0.176)	0.250 (0.162)

### Analytic Strategy

To examine the gender-emotion stereotypes, ANOVAs of error rates for various trials would be conducted. In the face recall task, gender-emotion stereotype is inferred when stereotypical mistakes are made with regard to responses about the emotional expression or gender of faces with various combinations of face types. Specifically, it is quantified as the difference in error rates between trials with stereotype-incongruent combinations (i.e., male faces with a fearful expression, or female faces with an angry expression) and stereotype-congruent combinations (i.e., male faces with an angry expression, or female faces with a fearful expression). This can be expressed by the following equation:

θ⁢(b⁢i⁢a⁢s)=P⁢(e⁢r⁢r⁢o⁢r|i⁢n⁢c⁢o⁢n⁢g⁢r⁢u⁢e⁢n⁢t)-P⁢(e⁢r⁢r⁢o⁢r|c⁢o⁢n⁢g⁢r⁢u⁢e⁢n⁢t).

To dissociate and evaluate the independent contribution of automatic and controlled processing to gender-emotion stereotypes, the revised PDP analysis was conducted (Payne, 2001; Huntsinger et al., 2009, 2010). The automatic and controlled processing responses can be respectively measured within the face recall task by comparing performance on distractor-congruent trials (i.e., information participants are instructed to identify about the target is congruent with the distractor) with distractor-incongruent trials (i.e., information participants are instructed to identify about the target is incongruent with the distractor). For distractor-congruent trials, inaccurate identification of an emotional expression or gender could only result from automatic processing in the absence of additional interference from the adjacent distractor. For distractor-incongruent trials, however, inaccurate identification could result from automatic processing or controlled processing. For example, both automatically activated stereotype linking men with angry expressions and illusory conjunction with the emotional expression of the adjacent distractor could lead to an incorrect response when the target is the emotional expression of fear on a male face in distractor-incongruent trials.

Specifically, in congruent trials, stereotypical mistakes could be driven only by automatic processing (S_aut_), expressed by the following equation:

θ(bias|same)=S.aut

In incongruent trials, stereotypical mistakes could be driven by either automatic processing (S_aut_) or controlled processing (S_con_) after controlling for the automatic stereotypical responses, expressed by the following equation:

θ(bias|different)=S+autS(1-S)autc⁢o⁢n.

Based on the equations above, we can estimate the automatic and controlled processing algebraically:

S=autθ(bias|same);

S=con(θ(bias|different)-θ(bias|same))/(1-θ(bias|same)).

## Results

Descriptive statistics (means and standard deviations) of error rates for every given type of target face when paired with different distractors are presented in Table 1. As expected, the overall error rates of emotion-recall trials were significantly higher than overall error rates of gender-recall trials [*t*(56) = 7.22, *p* < 0.001, *d* = 1.36], and the reaction times of correct answered trials display the similar pattern of difference [*t*(56) = 6.47, *p* < 0.001, *d* = 1.22], indicating that identifying the gender information may have been relatively easy and gender identification may take precedence over emotion identification.

**FIGURE 2:**
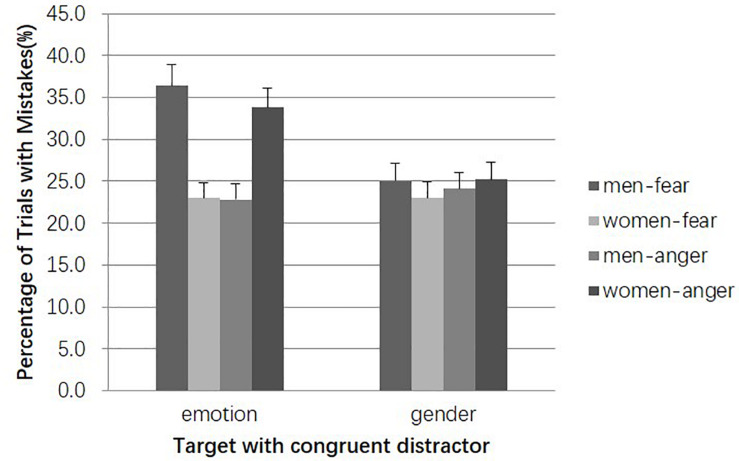
Error rates in distractor-congruent condition. Error bars represent standard errors.

Comparing the error rates for trials in distractor-congruent condition would reflect misperception with no interference information (see Figure 2). For trials in which emotion was recalled, a two-way repeated measures ANOVA with Target Gender (woman, man) and Target Emotion (anger, fear) revealed a significant interaction, *F*(1, 56) = 25.93, *p* < 0.001, η*_p_*^2^ = 0.32, without main effects for Target Gender or Emotion (*F*s < 1). Specific comparisons showed that the error rates for fearful male faces were significantly higher than those for fearful female faces [*t*(56) = 4.32, *p* < 0.001, *d* = 0.82] and angry male faces [*t*(56) = 3.95, *p* < 0.001, *d* = 0.75], indicating that male faces were more likely to be associated with an angry expression, as predicted. The hypothesis that it was more likely for female faces to be associated with a fearful expression was supported as well. The error rates for angry female faces were significantly higher than those for angry male faces [*t*(56) = 3.59, *p* < 0.01, *d* = 0.68] and fearful female faces [*t*(56) = 3.59, *p* < 0.01, *d* = 0.68]. However, there was no significant difference in the comparison among all types above in the trials in which gender was recalled (*p*s > 0.44). This indicates that the identification of gender information from faces was not affected by stereotype, thus further confirming the priming effect of gender on emotion.

**FIGURE 3:**
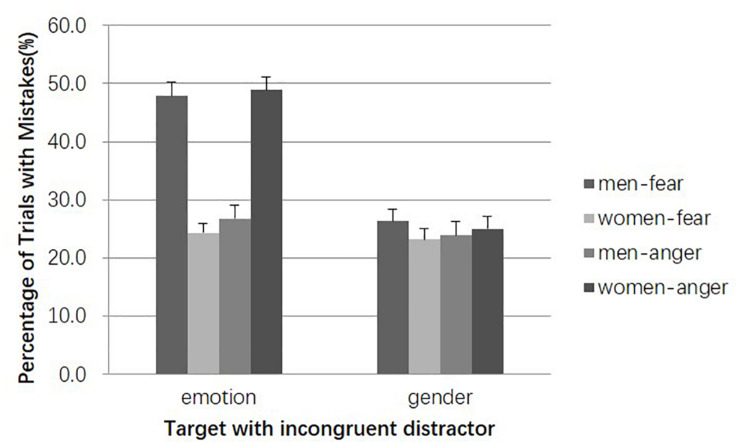
Error rates in distractor-incongruent condition. Error bars represent standard errors.

Comparing the error rates for trials in distractor-incongruent condition could allow for the effect of interference information (see Figure 3). A Target Gender (woman, man) × Target Emotion (anger, fear) ANOVA for trials in which emotion was recalled produced no significant main effects (*F*s < 1) but did demonstrate a predicted significant 2-way interaction, *F*(1, 56) = 84.17, *p* < 0.001, η*_p_*^2^ = 0.60. More specifically, paired samples *t*-tests revealed that the error rates for fearful male faces were significantly higher than those for fearful female faces [*t*(56) = 7.61, *p* < 0.001, *d* = 1.44] and angry male faces [*t*(56) = 5.47, *p* < 0.001, *d* = 1.03], and the error rates for angry female faces were significantly higher than those for angry male faces [*t*(56) = 7.08, *p* < 0.001, *d* = 1.34] and fearful female faces [*t*(56) = 7.93, *p* < 0.001, *d* = 1.50]. As found in the distractor-congruent trials, the connection between gender and emotion in the emotion identification trials also appeared when the emotional expressions of the distractors were incongruent with those of the targets and even had a much stronger effect. The results suggest that there may exist a controlled processing pathway in addition to the general tendency of automatic gender-emotion stereotype. Additionally, Target Gender and Target Emotion did not have any main effects or an interaction for trials in which gender was recalled (*p*s > 0.27).

We then used PDP analysis to dissociate the independent effects of dual-processing pathways on stereotypical perception tendencies regarding emotional expressions. The automatic (S_aut_) and controlled processing (S_con_) of gender-emotion stereotypes were obtained by the following calculations:

S=autθ(bias|same);

S=con(θ(bias|different)-θ(bias|same))/(1-θ(bias|same)).

The single-sample *t*-test for S_aut_ was significantly greater than zero, *t*(56) = 5.09, *p* < 0.001, *d* = 0.96. For S_con_, the result of the *t*-test was more moderate but still statistically significant, *t*(56) = 2.43, *p* < 0.05, *d* = 0.46, indicating that stereotype may include not only automatic processing but also an independent contribution from the controlled processing pathway.

## Discussion

### Stereotypical Connection Between Gender and Emotion

The present study demonstrates that the perception and identification of emotion information on human faces have a bias toward gender-related stereotypes, which tend to associate male faces with angry expressions and female faces with fearful expressions. Considering the differences reflected in gender roles, these perceptual biases may have developed to eliminate potential threats and seize beneficial opportunities as a consequence of evolution (Miller et al., 2010). Since men seem to be more aggressive and capable of harming others in individuals’ general experience, the bias to perceive a man as angry serves a protective function (Sell et al., 2009). In contrast, women seem to be more vulnerable and frailer, thus fear is believed to occur more frequently in women.

While obtaining gender-related information from the face image, which acted as the information carrier used in this study, we would inevitably extract relevant features of facial structure, such as dominance and trustworthiness (Oh et al., 2019), with dominance more correlated with masculinity and trustworthiness more correlated with femininity. It has been shown by previous studies that dominance and trustworthiness could also trigger differentiated emotional perception, and further explain the stereotypical connection between gender and emotion. Specifically, more dominant-looking faces (i.e., those appearing more mature) were more readily perceived as angry compared to neotenous faces, which were more readily perceived as fearful (Sacco and Hugenberg, 2009), and trustworthy faces who expressed anger were perceived as less angry than untrustworthy faces (Oosterhof and Todorov, 2009).

From a developmental perspective, these stereotypical expectations begin to develop from infancy through a feedback loop in which caregivers, constrained by socializing rules for different genders in certain cultures, interpret and respond to the expressions of infants (Holodynski, 2013). Therefore, cultural norm may also play a unique role in the formation of stereotypes in specific cultural backgrounds or societies (Nelson et al., 2012; Moran et al., 2013). Especially considering the significant cross-cultural differences in emotional expression and experience (Harell et al., 2015; Lim, 2016), it is necessary to limit the cultural context to examine the stereotypical connection between gender and emotion. The present study was conducted with undergraduates who are all native Chinese of Asian descent to hold the culture constant, which was often ignored in previous researches and in turn led to variability in results. Similar findings of stereotypical response tendencies on face recognition have also revealed in prior studies with Western samples (Adams et al., 2012; Neel et al., 2012), indicating that the gender-emotion stereotype is ubiquitous in different cultures.

### Asymmetry of Gender-Emotion Stereotype

However, the gender-emotion stereotype does not occur in all conditions of facial perception. Only when individuals were instructed to identify emotional expressions did this stereotype come into effect, but it disappeared in identification of gender information. Although similar asymmetrical connection between gender and emotion in visual processing has been found in previous studies (Atkinson et al., 2005; Karnadewi and Lipp, 2011), a longstanding controversy over whether facial features are processed independently or interdependently has led to different explanations for its presence.

According to the Bruce-Young Model (for a review, see Young and Bruce, 2011), as a particularly comprehensive framework for understanding face recognition from the perspective of independent processing, the visual processing of facial information contains multiple pathways that are independent of each other and different in processing priority. More specifically, people preferentially process the most intuitive and basic coding information (e.g., graphic code, structure code) and subsequently encode more complex information. The visual-semantic code that includes gender information takes precedence over the expression code in processing priorities. Thus, when both gender and emotion information must be simultaneously processed, people first establish the gender impression, followed by the emotion impression. Namely, the reason why gender perception may not be affected by gender-emotion connections is that gender information is processed more quickly and easily than emotion identification, whereas emotion information processing is affected by this perceptual bias after gender information is obtained.

From an interdependent processing perspective, the processing of facial features may run as a holistic perception with shared underlying neural processes (Bestelmeyer et al., 2010), suggesting that the encoding of gender and emotional expression have no differences in processing priorities. However, empirical findings have shown that the expected perceptual aftereffect of emotional expression was not observed after adapting to the angry male face, which acted as a compatible facial combination with gender-emotion stereotype, resulted by either increased salience or weakened adaption (Harris and Ciaramitaro, 2016). Both mechanisms indicate a special status of angry male face in emotion perception, further implying that certain combinations of facial features may give rise to biased processing of emotion information. In addition, compared to the gender information generally regarded as invariant physical cues, emotion information is more dynamic and difficult to conceptualize. It has been demonstrated that the neural mechanisms for invariant and dynamic facial feature processing may be shared during perceptual encoding, but separate during recognition and decision making (Pallett and Meng, 2013). Therefore, it can be deduced that the identification of emotion, with higher uncertainty of encoding cues, may be more susceptible to the top-down influences including stereotypes.

### Dual-Processing Mechanism of Gender-Emotion Stereotype

Finally, and definitely most importantly, the present study also confirmed the dual-processing mechanism of gender-emotion stereotype. Compared with the distractor-congruent condition, the gender-emotion stereotypes had a larger effect when the emotion information of the target was incongruent with the distractor, which affected the emotion perception via an additional interference effect. More precisely, providing an interference stimulus that conflicted with the target emotion increased the error rates for emotion perception, but only for trials with the stereotype-incongruent combinations between gender and emotion (i.e., man-fear, woman-anger), whereas the error rates for faces compatible with stereotype (i.e., man-anger, woman-fear) did not increase with the appearance of interference information (*p*s > 0.20), suggesting that the bottom-up processing only affect emotion identification by grabbing the emotion cues stereotypically corresponding to target gender. When the emotion information of a face is stereotypically associated with its gender information, the emotion identification would not be affected by another emotion on the adjacent face.

Findings from the revised PDP analysis showed that the stereotypical response is a consequence of both automatic and controlled processing, which is consistent with previous studies (Huntsinger et al., 2009, 2010). From an affordance-based perspective, cognitive processing is the result of interactions between people and the environment, referring to generalized external stimuli, including contexts, media, and other substance objects (Gibson, 1979). The extent of affordance depends on how well the information obtained from the environment is connected with the existing experience stored in the mind. When external information is consistent with our existing experience, it gets better attention and processing. In this study, the emotion of the distractor represents environmental information, and it had the largest impact on emotion perception when it was incongruent with the target and consistent with the gender-emotion stereotype for the target gender.

Another comprehensive model that contributes to understand the dual-processing mechanism is the dynamic interactive theory, which interprets individual perception as a dynamical system involving continuous interaction between low-level sensory perception and high-order social cognition (Freeman and Ambady, 2011). As a high-order social cognitive process, stereotype can either directly affect the retrieval of facial features or in turn be modified by visual cues in the background. And this interactive process also has a clear neural network basis (Freeman and Johnson, 2016), including the fusiform gyrus (FG) involved in the visual processing of faces, the anterior temporal lobe (ATL) retrieving the social-conceptual associations including stereotypes related to perceived characteristics, and the orbitofrontal cortex (OFC) integrating information to implement top-down visual predictions, which would further modulate FG’s representations of faces.

### Implications and Limitations

Taken together, the present study examined the multiple connections between gender and emotion in face recognition, contributing to future research on theory and methodology. We effectively dissociated the dual-processing of gender-emotion stereotypes, confirming the independent effect of a controlled processing pathway on stereotypical response. Moreover, the revised PDP analysis can be used to discriminate the function between automatic and controlled components in gender-emotion research or combined with other related paradigms. Still, some issues also remain that have not been addressed and should be considered in future research.

The main limitation of the current work is that we investigated the connection between gender and emotion only based on the manipulation of face images. As mentioned above, facial structure contains a wealth of information in visual processing, some of which may be responsible for the perceptual biases of emotion found in this study. In addition to dominance and trustworthiness, there are also other facial features that have an impact on emotional perception, such as facial width-to-height ratio (fWHR), which is not necessarily influenced by gender (Deska et al., 2018). As questioned by Brody et al. (2016) in their review, it is not clear in such face recognition studies whether the perceptual bias is caused by the base-rate beliefs about emotional experience/expression for different genders or certain visual cues for obtaining selective attention. We could not completely rule out other explanations for our findings without an effective control for relevant features of facial structure. Thus, more convincing evidence of this point would be to further manipulate facial features or combine with other materials and corresponding paradigm (e.g., semantic objects).

Another confounding factor that needs to be controlled is the emotional state of the perceivers. Previous study has confirmed that perceptual biases of emotional expressions for faces of different genders depends on the participants’ current state affect (Harris et al., 2016), suggesting a moderating effect of current emotional state on gender-emotion stereotypes. The corollary is that the changes of emotion caused by the experimental manipulations may also limit the explanations for our findings. As presented in procedure, information processing in distractor-incongruent condition is obviously more difficult than in distractor-congruent condition, in which the perceivers would experience a stronger uncertainty. It has also been found that the increased uncertainty would be accompanied by an enhancement in insecurity (Aurigemma and Mattson, 2018), which make the perceivers more sensitive to possible threat signals or encourage them to choose processing strategies with a lower cognitive load. Although similar paradigms have been widely used in face recognition studies (e.g., Neel et al., 2012), little attention has been paid to the emotions experienced in the experiment and their effects on cognitive responses, which need to be further explored in detail.

## Data Availability Statement

The datasets generated for this study are available on request to the corresponding author.

## Ethics Statement

The studies involving human participants were reviewed and approved by the Research Ethics Committee of Beijing Normal University. The patients/participants provided their written informed consent to participate in this study.

## Author Contributions

WZ contributed to stimulus generation, experimental design, data analysis and interpretation, and writing of the manuscript. PF contributed to experimental design, data interpretation, and writing of the manuscript as co-first author. HX and YM contributed to data collection, stimulus presentation, and data analysis. MY contributed to data interpretation and writing of the manuscript.

## Conflict of Interest

The authors declare that the research was conducted in the absence of any commercial or financial relationships that could be construed as a potential conflict of interest.

## Footnotes

1 *A priori* sample size was estimated with settings of the effect size (0.30), α error probability (0.05), and β error probability (0.20) at a moderate level, obtaining that the required sample size was calculated to be no less than 46.

2 Participants were also asked to rate the abnormality and attractiveness of faces respectively on a 7-point scale, with higher scores indicating that a certain face was more abnormal and attractive. All the retained stimuli used in the experiment should be no more than 5 on abnormality and between 3 and 5 on attractiveness.
