# Supplementary figures and images for: Not Only Top-Down: The Dual-Processing of Gender-Emotion Stereotypes
Source: Front Psychol. 2020 May 26;11:1042. doi: 10.3389/fpsyg.2020.01042 (PMC7264380; doi:10.3389/fpsyg.2020.01042)

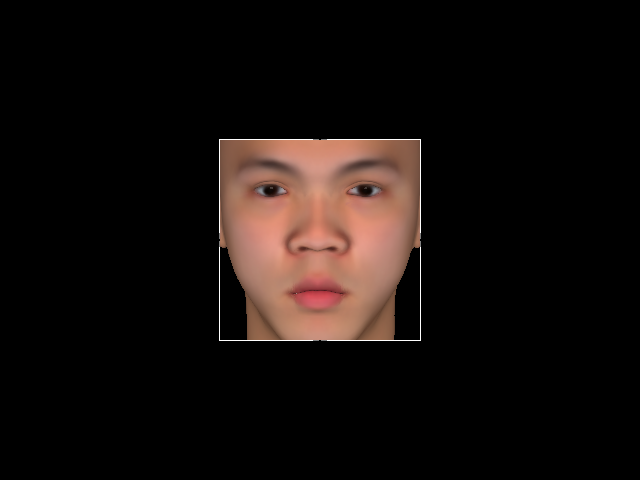

Supplement: Supplementary file 2 [file Data_Sheet_2.ZIP › í╛Materialsí┐/Material evaluation (Neutral)/01001.bmp]

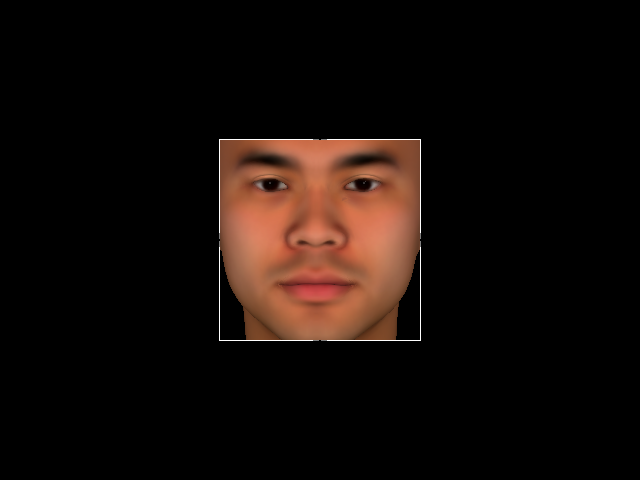

Supplement: Supplementary file 2 [file Data_Sheet_2.ZIP › í╛Materialsí┐/Material evaluation (Neutral)/01101.bmp]

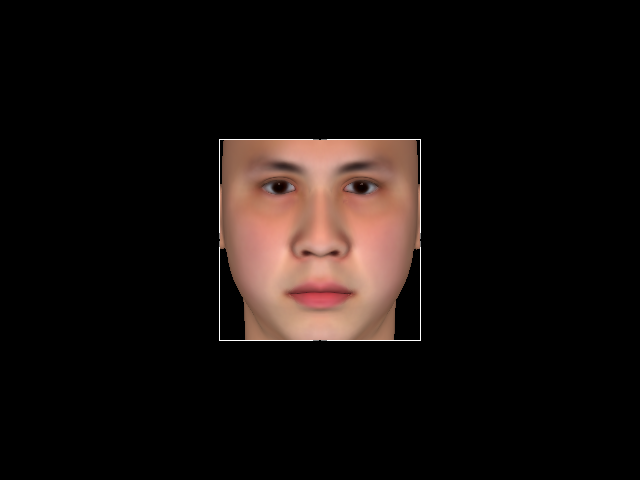

Supplement: Supplementary file 2 [file Data_Sheet_2.ZIP › í╛Materialsí┐/Material evaluation (Neutral)/01201.bmp]

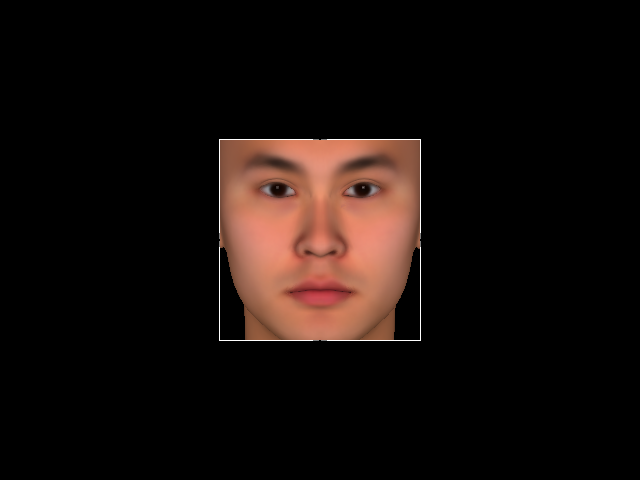

Supplement: Supplementary file 2 [file Data_Sheet_2.ZIP › í╛Materialsí┐/Material evaluation (Neutral)/01301.bmp]

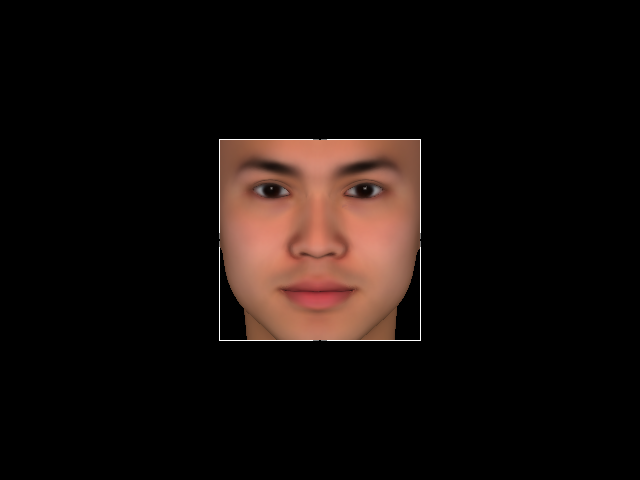

Supplement: Supplementary file 2 [file Data_Sheet_2.ZIP › í╛Materialsí┐/Material evaluation (Neutral)/01401.bmp]

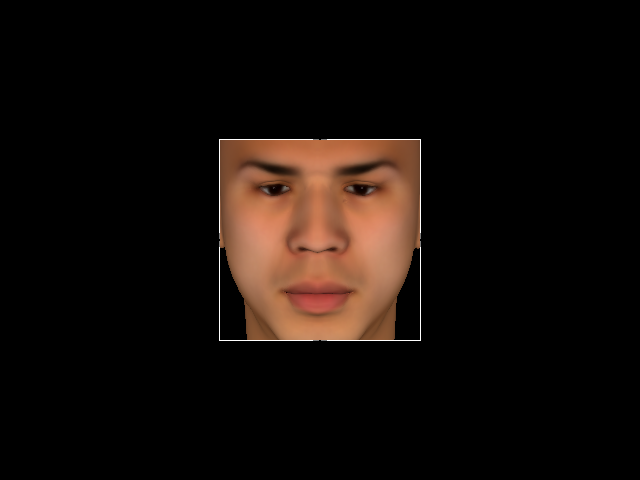

Supplement: Supplementary file 2 [file Data_Sheet_2.ZIP › í╛Materialsí┐/Material evaluation (Neutral)/01501.bmp]

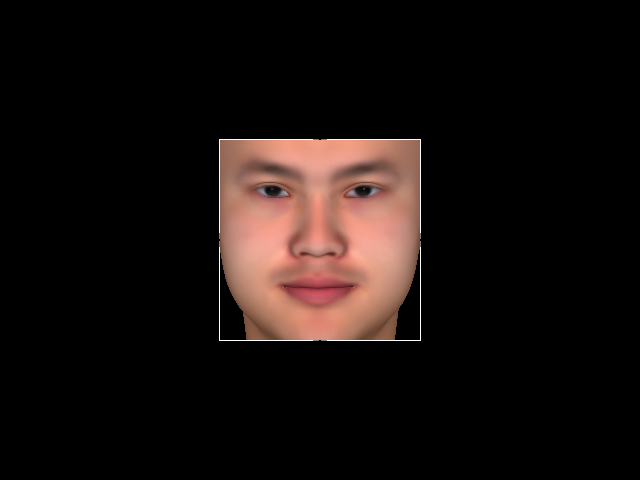

Supplement: Supplementary file 2 [file Data_Sheet_2.ZIP › í╛Materialsí┐/Material evaluation (Neutral)/01601.bmp]

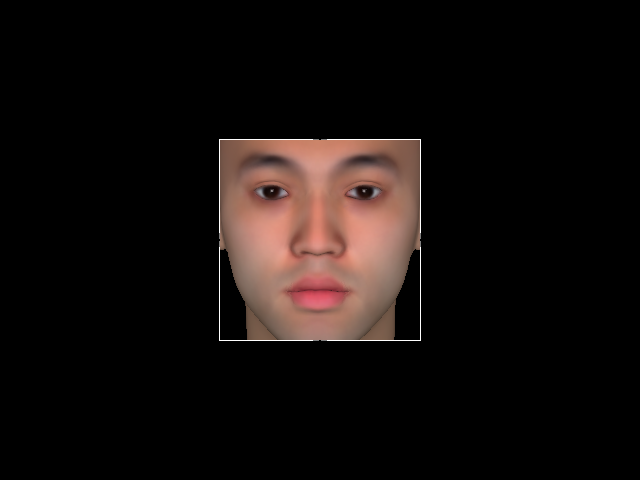

Supplement: Supplementary file 2 [file Data_Sheet_2.ZIP › í╛Materialsí┐/Material evaluation (Neutral)/01701.bmp]

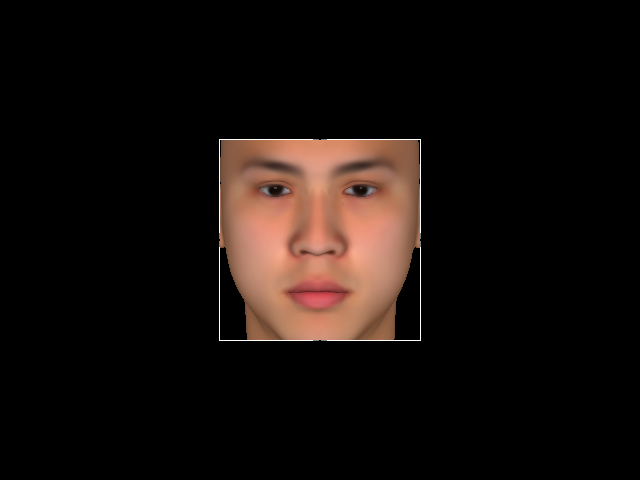

Supplement: Supplementary file 2 [file Data_Sheet_2.ZIP › í╛Materialsí┐/Material evaluation (Neutral)/01801.bmp]

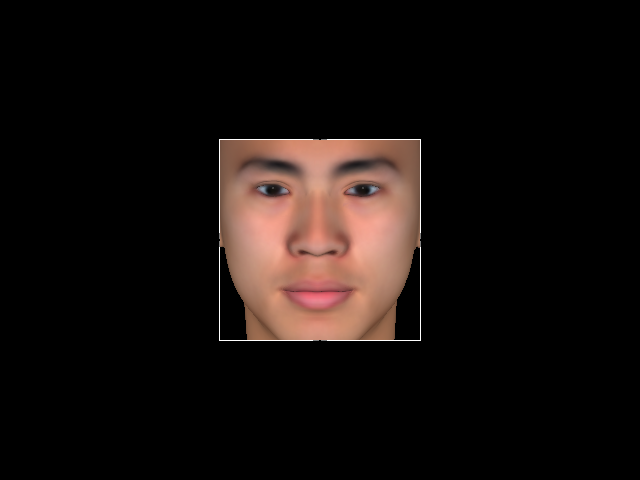

Supplement: Supplementary file 2 [file Data_Sheet_2.ZIP › í╛Materialsí┐/Material evaluation (Neutral)/01901.bmp]

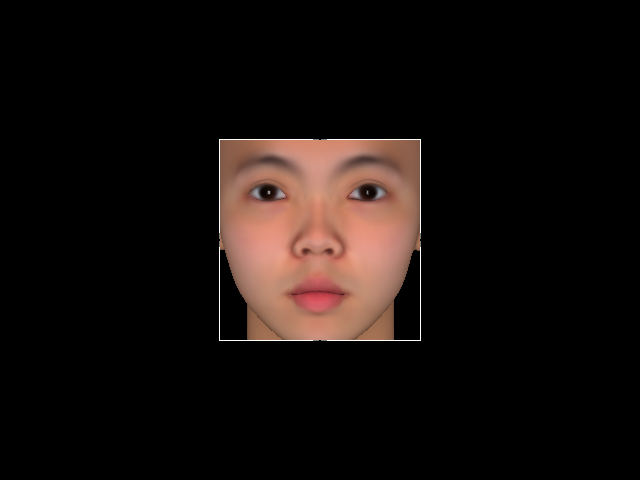

Supplement: Supplementary file 2 [file Data_Sheet_2.ZIP › í╛Materialsí┐/Material evaluation (Neutral)/02001.bmp]

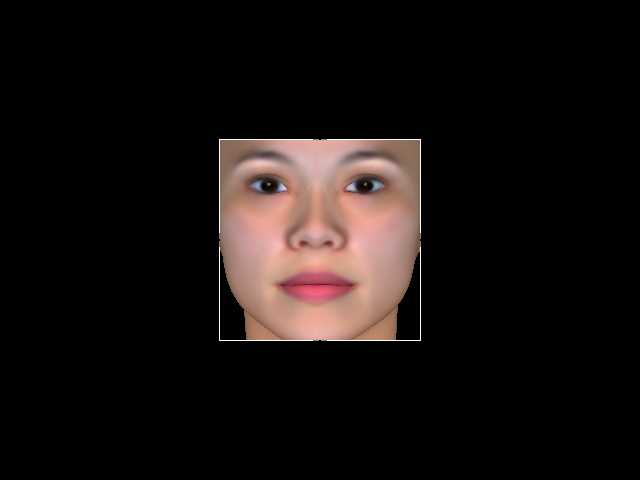

Supplement: Supplementary file 2 [file Data_Sheet_2.ZIP › í╛Materialsí┐/Material evaluation (Neutral)/02101.bmp]

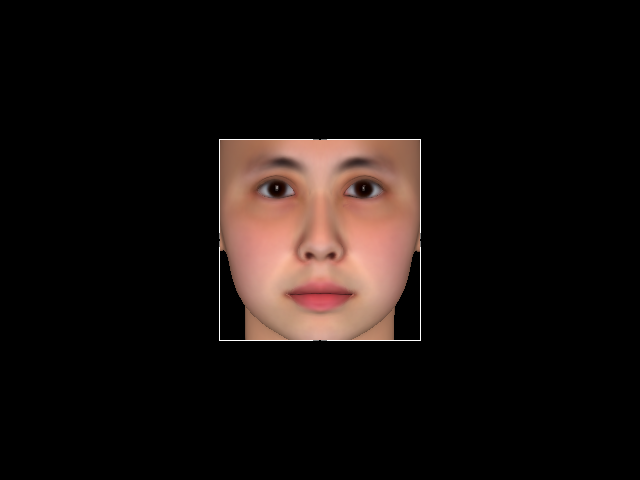

Supplement: Supplementary file 2 [file Data_Sheet_2.ZIP › í╛Materialsí┐/Material evaluation (Neutral)/02201.bmp]

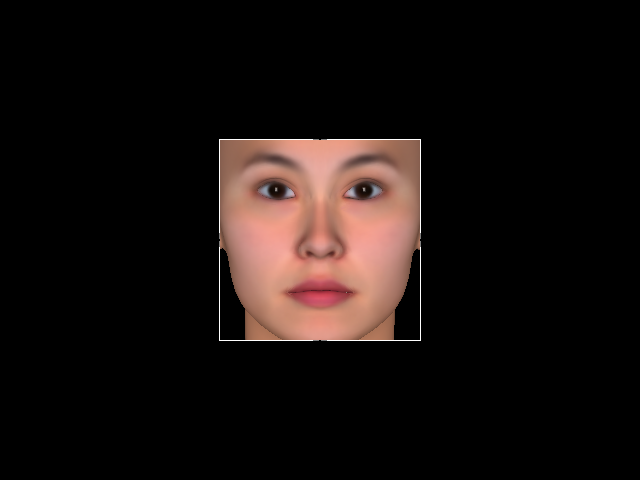

Supplement: Supplementary file 2 [file Data_Sheet_2.ZIP › í╛Materialsí┐/Material evaluation (Neutral)/02301.bmp]

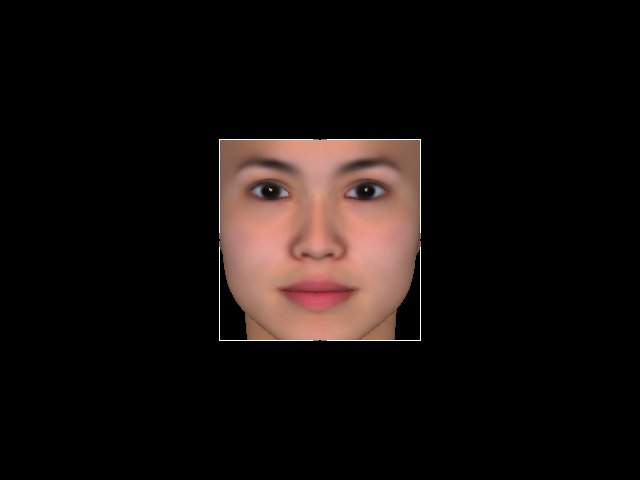

Supplement: Supplementary file 2 [file Data_Sheet_2.ZIP › í╛Materialsí┐/Material evaluation (Neutral)/02401.bmp]

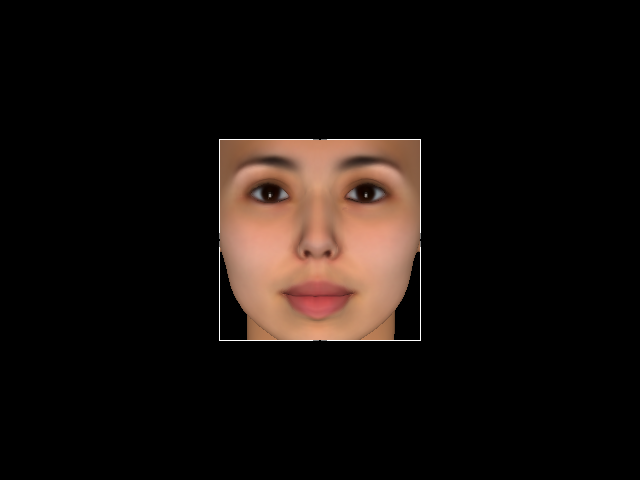

Supplement: Supplementary file 2 [file Data_Sheet_2.ZIP › í╛Materialsí┐/Material evaluation (Neutral)/02501.bmp]

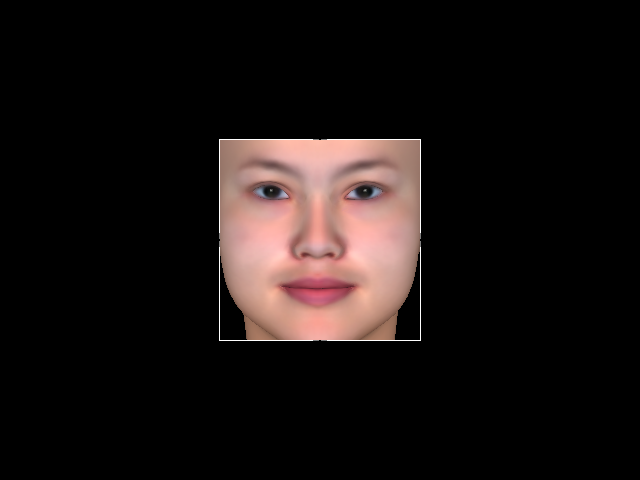

Supplement: Supplementary file 2 [file Data_Sheet_2.ZIP › í╛Materialsí┐/Material evaluation (Neutral)/02601.bmp]

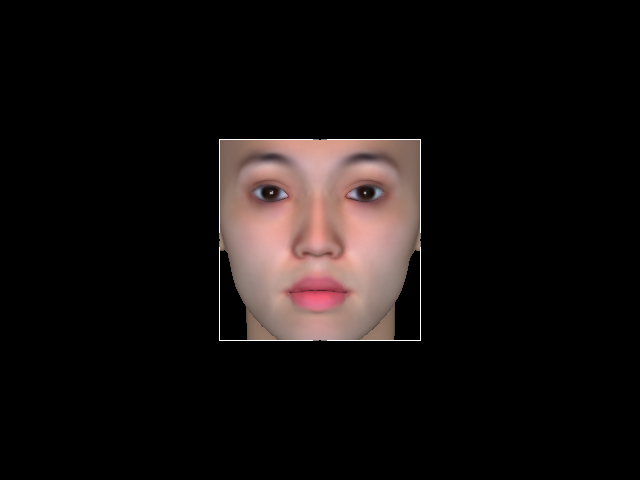

Supplement: Supplementary file 2 [file Data_Sheet_2.ZIP › í╛Materialsí┐/Material evaluation (Neutral)/02701.bmp]

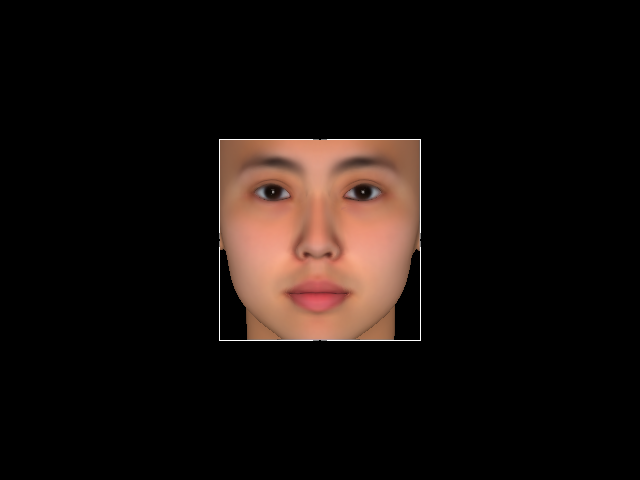

Supplement: Supplementary file 2 [file Data_Sheet_2.ZIP › í╛Materialsí┐/Material evaluation (Neutral)/02801.bmp]

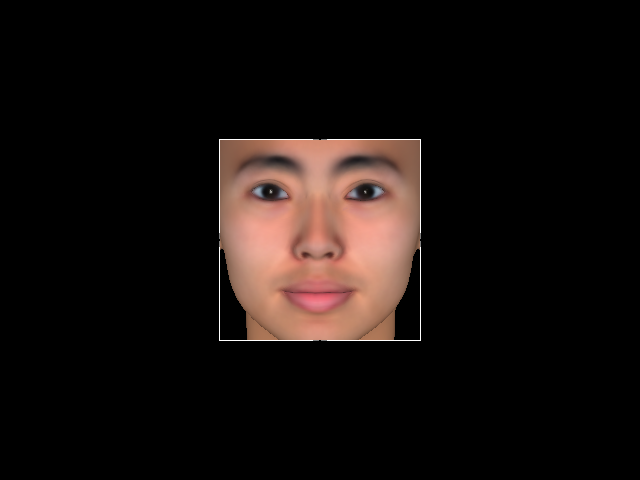

Supplement: Supplementary file 2 [file Data_Sheet_2.ZIP › í╛Materialsí┐/Material evaluation (Neutral)/02901.bmp]

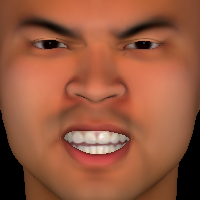

Supplement: Supplementary file 2 [file Data_Sheet_2.ZIP › í╛Materialsí┐/Target stimuliú¿Fearful & Angryú⌐/Critical stimuli/1102.bmp]

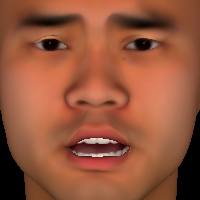

Supplement: Supplementary file 2 [file Data_Sheet_2.ZIP › í╛Materialsí┐/Target stimuliú¿Fearful & Angryú⌐/Critical stimuli/1103.bmp]

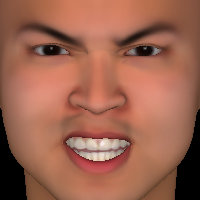

Supplement: Supplementary file 2 [file Data_Sheet_2.ZIP › í╛Materialsí┐/Target stimuliú¿Fearful & Angryú⌐/Critical stimuli/1402.bmp]

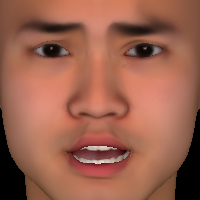

Supplement: Supplementary file 2 [file Data_Sheet_2.ZIP › í╛Materialsí┐/Target stimuliú¿Fearful & Angryú⌐/Critical stimuli/1403.bmp]

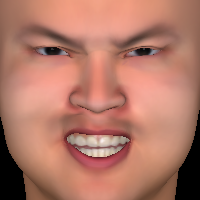

Supplement: Supplementary file 2 [file Data_Sheet_2.ZIP › í╛Materialsí┐/Target stimuliú¿Fearful & Angryú⌐/Critical stimuli/1602.bmp]

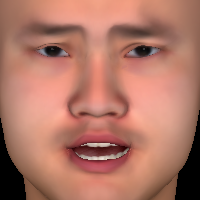

Supplement: Supplementary file 2 [file Data_Sheet_2.ZIP › í╛Materialsí┐/Target stimuliú¿Fearful & Angryú⌐/Critical stimuli/1603.bmp]

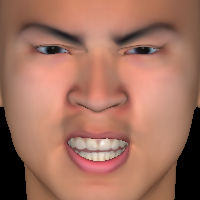

Supplement: Supplementary file 2 [file Data_Sheet_2.ZIP › í╛Materialsí┐/Target stimuliú¿Fearful & Angryú⌐/Critical stimuli/1902.bmp]

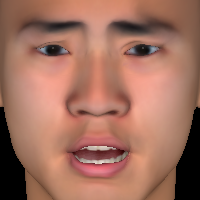

Supplement: Supplementary file 2 [file Data_Sheet_2.ZIP › í╛Materialsí┐/Target stimuliú¿Fearful & Angryú⌐/Critical stimuli/1903.bmp]

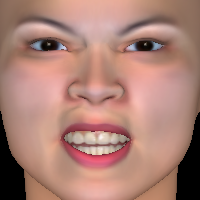

Supplement: Supplementary file 2 [file Data_Sheet_2.ZIP › í╛Materialsí┐/Target stimuliú¿Fearful & Angryú⌐/Critical stimuli/2102.bmp]

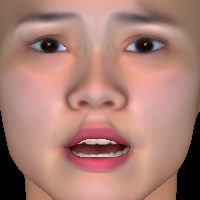

Supplement: Supplementary file 2 [file Data_Sheet_2.ZIP › í╛Materialsí┐/Target stimuliú¿Fearful & Angryú⌐/Critical stimuli/2103.bmp]

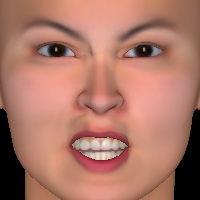

Supplement: Supplementary file 2 [file Data_Sheet_2.ZIP › í╛Materialsí┐/Target stimuliú¿Fearful & Angryú⌐/Critical stimuli/2302.bmp]

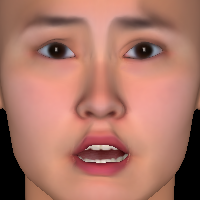

Supplement: Supplementary file 2 [file Data_Sheet_2.ZIP › í╛Materialsí┐/Target stimuliú¿Fearful & Angryú⌐/Critical stimuli/2303.bmp]

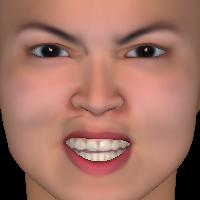

Supplement: Supplementary file 2 [file Data_Sheet_2.ZIP › í╛Materialsí┐/Target stimuliú¿Fearful & Angryú⌐/Critical stimuli/2402.bmp]

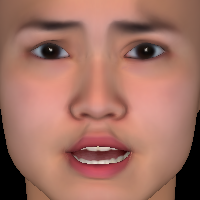

Supplement: Supplementary file 2 [file Data_Sheet_2.ZIP › í╛Materialsí┐/Target stimuliú¿Fearful & Angryú⌐/Critical stimuli/2403.bmp]

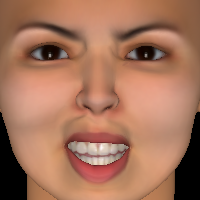

Supplement: Supplementary file 2 [file Data_Sheet_2.ZIP › í╛Materialsí┐/Target stimuliú¿Fearful & Angryú⌐/Critical stimuli/2502.bmp]

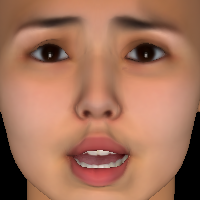

Supplement: Supplementary file 2 [file Data_Sheet_2.ZIP › í╛Materialsí┐/Target stimuliú¿Fearful & Angryú⌐/Critical stimuli/2503.bmp]

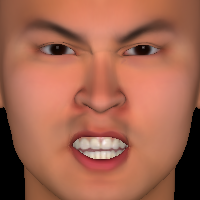

Supplement: Supplementary file 2 [file Data_Sheet_2.ZIP › í╛Materialsí┐/Target stimuliú¿Fearful & Angryú⌐/Practice stimuli/1302.bmp]

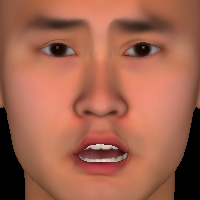

Supplement: Supplementary file 2 [file Data_Sheet_2.ZIP › í╛Materialsí┐/Target stimuliú¿Fearful & Angryú⌐/Practice stimuli/1303.bmp]

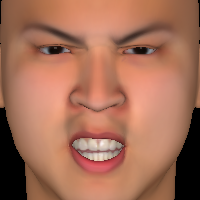

Supplement: Supplementary file 2 [file Data_Sheet_2.ZIP › í╛Materialsí┐/Target stimuliú¿Fearful & Angryú⌐/Practice stimuli/1802.bmp]

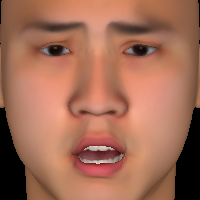

Supplement: Supplementary file 2 [file Data_Sheet_2.ZIP › í╛Materialsí┐/Target stimuliú¿Fearful & Angryú⌐/Practice stimuli/1803.bmp]

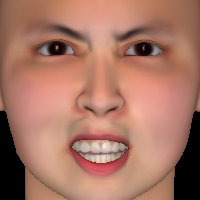

Supplement: Supplementary file 2 [file Data_Sheet_2.ZIP › í╛Materialsí┐/Target stimuliú¿Fearful & Angryú⌐/Practice stimuli/2202.bmp]

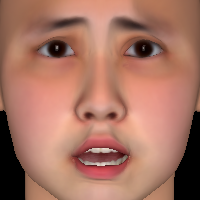

Supplement: Supplementary file 2 [file Data_Sheet_2.ZIP › í╛Materialsí┐/Target stimuliú¿Fearful & Angryú⌐/Practice stimuli/2203.bmp]

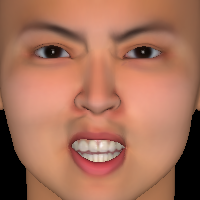

Supplement: Supplementary file 2 [file Data_Sheet_2.ZIP › í╛Materialsí┐/Target stimuliú¿Fearful & Angryú⌐/Practice stimuli/2802.bmp]

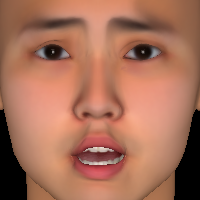

Supplement: Supplementary file 2 [file Data_Sheet_2.ZIP › í╛Materialsí┐/Target stimuliú¿Fearful & Angryú⌐/Practice stimuli/2803.bmp]
